# Risk factors for febrile urinary tract infection in boys with posterior urethral valves

**DOI:** 10.3389/fped.2022.971662

**Published:** 2022-09-14

**Authors:** Luke Harper, Nathalie Botto, Matthieu Peycelon, Jean-Luc Michel, Marc-David Leclair, Sarah Garnier, Pauline Clermidi, Alexis P. Arnaud, Anne-Laure Dariel, Eric Dobremez, Alice Faure, Laurent Fourcade, Nadia Boudaoud, Yann Chaussy, Fideline Collin, Laetitia Huiart, Cyril Ferdynus, Valery Bocquet, Frederique Sauvat

**Affiliations:** ^1^Department of Pediatric Surgery, CHU de La Réunion, Saint-Denis, France; ^2^Department of Pediatric Surgery and Urology, University Hospital Pellegrin-Enfants, CHU de Bordeaux, Bordeaux, France; ^3^Department of Pediatric Surgery and Urology, APHP, Hôpital Necker, Paris, France; ^4^Department of Pediatric Urology, University Hospital Robert Debre, APHP, Centre de Référence des Malformations Rares des Voies Urinaires (MARVU), University of Paris, Paris, France; ^5^Department of Pediatric Surgery and Urology, Children's University Hospital, CHU de Nantes, Nantes, France; ^6^Department of Pediatric Surgery and Urology, Lapeyronie University Hospital, CHU de Montpellier, Montpellier, France; ^7^Department of Pediatric Surgery, Armand Trousseau Children's University Hospital, Paris, France; ^8^Department of Pediatric Surgery, Rennes University Hospital, CHU de Rennes, Rennes, France; ^9^Department of Pediatric Surgery, North and Timone Children's Hospital, Assistance Publique Hopitaux de Marseille, Aix-Marseille Université, Marseille, France; ^10^Department of Pediatric Surgery, University Hospital, CHU de Limoges, Limoges, France; ^11^Department of Pediatric Surgery, Reims University Hospital, Reims, France; ^12^Department of Pediatric Surgery, Besançon University Hospital, CHU de Besançon, Besançon, France; ^13^Unité de Soutien Méthodologique, CHU de La Réunion, Saint-Denis, France; ^14^Clinical Research Department, INSERM, CIC1410, CHU de La Réunion, Saint-Pierre, France

**Keywords:** posterior urethral valves, urinary tract abnormalities, infection, risk factors, reflux

## Abstract

**Objective:**

Boys with posterior urethral valves (PUV) present an increased risk of febrile urinary tract infection (fUTI). Identifying specific risk factors could allow for tailoring UTI prevention. The aim of this study was to use the data from the CIRCUP randomized controlled trial data to identify patient characteristics associated with a higher risk of fUTI.

**Patients and methods:**

We performed a secondary analysis of the data from the CIRCUP randomized trial which included boys with PUV, randomized to circumcision and antibiotic prophylaxis vs. antibiotic prophylaxis alone and followed for 2 years. There was only 1 episode of fUTI in the circumcision group vs. 17 in the uncircumcised group. We therefore only studied the antibiotic prophylaxis alone group and compared age at prenatal diagnosis, size and weight at birth, presence of dilating VUR at diagnosis, abnormal DMSA scan at 2 months, and nadir creatinine between children who presented a fUTI and those who did not, as well as age at first episode of fUTI.

**Results:**

The study group consisted of 42 patients of which 17 presented at least on fUTI. Presence of dilating VUR was significantly associated with risk of fUTI (*p* = 0.03), OR: 6 [CI 95% = (1.13–27.52)]. None of the other parameters were associated with increased risk of fUTI. We observed three distinct time periods for presenting a fUTI with a decrease in infection rate after the first 40 days of life, then at 240 days of life.

**Conclusion:**

In boys with PUV, presence of high-grade VUR is associated with a higher risk of presenting a fUTI. The rate of febrile UTIs seems to decrease after 9 months.

## Introduction

Posterior urethral valves (PUV) represent the most common cause of Lower Urinary Tract Obstruction (LUTO) in boys. They affect 1:4000–1:25,000 births, and cause increased intravesical pressure during fetal kidney development resulting in various degrees of kidney and bladder impairment (1, 2). In spite of postnatal management, these patients present an increased risk of febrile urinary tract infection (fUTI) which puts these children's kidneys at particular risk of ongoing damage (3). However, the clinical spectrum of PUV is very wide; meaning blanket recommendations for prevention of fUTI can be inadequate. Identifying specific risk factors for fUTI could allow for tailoring UTI prevention.

The CIRCUP randomized controlled trial (Clinicaltrials registration number: NCT01537601) was conducted to determine whether circumcision decreased the risk of febrile UTI in boys with PUV (4). The outcome showed that circumcision significantly decreases the risk of fUTI. The aim of this study was to perform a secondary analysis of the data from this trial to identify patient characteristics associated with a higher risk of fUTI.

## Patients and methods

The CIRCUP trial included children with PUV confirmed by voiding cystogram performed before 28 days of life, who were included at the time of valve resection and randomized to circumcision and antibiotic prophylaxis (49 patients) or no circumcision and antibiotic prophylaxis (42 patients) and followed prospectively for 2 years. Antibiotic prophylaxis consisted of cefaclor 10 mg/kg/day for the 1 month of life followed by cotrimoxazole 15 mg/kg/day up to the age of two.

The diagnosis of febrile UTI was defined as fever (>38.5°C) with evidence of pyuria and culture-proven infection on urinalysis, obtained by urethral catheterization or suprapubic aspiration, as well as biological signs of inflammation (defined as presence of leukocytosis, and a CRP level > 40 mg/L and/or procalcitonin > 0.5 ng/ml). We also included episodes of fUTI, where the urine samples were collected by clean catch and not by urethral catheterization or suprapubic aspiration but were validated by a scientific committee on the basis of the clinical and biological data.

A control cystogram was performed 3 months after valve resection in all patients to confirm absence of residual leaflets. A technetium-99 m–labeled dimercaptosuccinic acid (DMSA) scan was performed at 2 months. Patients were followed for 2 years. Adherence to antibiotic prophylaxis was evaluated using a questionnaire given to the parents during each outpatient visit.

There was only 1 episode of fUTI in the circumcision group (1/49) vs. 17 in the uncircumcised group (17/42). For obvious statistical reasons we could only therefore compare the characteristics of children with or without fUTI in the uncircumcised group. For this study we hence only used the control group. We compared age, size and weight at birth, age at diagnosis (second or third trimester), presence of VUR at diagnosis, abnormal DMSA scan at 2 months of life, and nadir creatinine (lowest creatinine within the 1st year of age) between children who presented a fUTI and those who did not. We also looked at age of presentation of the first episode of fUTI.

Qualitative variables were described in terms of number and percentage. Quantitative variables were described in terms of number, mean and standard deviation, or number, median and interquartile range if the conditions of application are not met. Comparisons of quantitative variables were made using the Student or Wilcoxon test depending on the application conditions. The qualitative variables were compared using the Chi^2^ test or Fisher's exact test depending on the application conditions.

In order to measure the effect on the presence of febrile urinary tract infections, a logistic regression was carried out with the explanatory variables: high-grade reflux of the patient, age, abnormal DMSA, weight, and height at the birth of the child. We selected for the multivariate analysis those variables whose *P*-value was < 0.10 in the univariate model. A stepwise selection model was then performed to detect the predictors of the outcome.

All analyzes were performed with SAS software (SAS System for Windows, version 9.4; SAS Institute Inc., Cary, NC). A *p* < 0.05 in the bilateral case was considered statistically significant.

The data used in this study comes from the CIRCUP RCT which was approved by the Programme Hospitalier de Recherche Clinique 2012 of the French Ministry of Health and validated by the relevant ethics committee. All parents or legal guardians of the children provided written informed consent before enrollment (Clinicaltrials registration number: NCT01537601).

## Results

The study group therefore consisted of 42 uncircumcised boys with PUV of which 17 presented at least one fUTI. Their clinical characteristics are summarized in Table 1. During the 1st year, four children interrupted their follow-up. During the 2nd year, seven children interrupted their follow-up. At the end of the 2 years, 31 children had a complete follow-up. Adherence to antibiotic prophylaxis decreased progressively during follow-up. Seventy-four percent of parents continued to give antibiotic prophylaxis at 6 months, 66% continued at 1 year and 47% at 2 years. However, the children who presented fUTIs were not the ones who interrupted their antibiotic prophylaxis.

**Table 1:** Patient characteristics (*N* = 42).

**Patient characteristics**	**Total**
fUTI, *N* (%)	17 (40.5%)
Age at 1st fUTI (>6 months) (*N* = 17), *N* (%)	6 (35.3%)
Length at birth (*N* = 38), cm, median (Q1–Q3)	50 (48–51)
Weight at birth (*N* = 42), g, mean (Standard deviation)	3203 (529)
Age at diagnosis (*N* = 34), GA, median (Q1–Q3)	32 (24–33)
Presence of VUR, *N* (%)	25 (62.5%)
Dysplasia on DMSA, *N* (%)	20 (60.6%)
Nadir creatinin (≥75 mol/L), *N* (%)	5 (11.9%)

fUTI, febrile urinary tract infection; VUR, vesicoureteral reflux.

Regarding fUTI comparisons, there was no statistically significant difference between patients with fUTIs and those without in terms of: size at birth (*p* = 0.15), age at suspected diagnosis (*p* = 0.23), abnormal DMSA scan (*p* = 0.46), or nadir creatinine (*p* = 0.38). There was a statistically significant difference between the two groups with regard to birth weight (*p* = 0.02) and presence of VUR (*p* = 0.04; Table 2).

**Table 2:** Patient characteristics according to presence or absence of fUTI.

**Patient characteristics**	**fUTI** **(*N* = 17)**	**No fUTI** **(*N* = 25)**	***P*** **-value**
Length at birth, cm, median (Q1–Q3)	50 (48–51)	49 (47–50)	0.15 (WIL)
Weight at birth, gram, mean (Standard deviation)	3,430 (420)	3,049 (547)	0.02
Age at diagnosis, GA, median (Q1-Q3)	28 (22–34)	32 (31–33)	0.23 (WIL)
Presence of VUR, *N* (%)	13 (81.2%)	12 (50.0%)	0.04
Dysplasia on DMSA, *N* (%)	8 (72.7%)	12 (54.5%)	0.46 (FE)
Nadir creatinin (≥75 mol/L), *N* (%)	3 (17.6%)	2 (8.0%)	0.38 (FE)

WIL, Wilcoxon; FE, Fisher exact test.

Three variables were selected for the multivariable analysis (Table 3): birth weight, age at diagnosis, and presence of VUR. There was a statistically significant effect of the presence of VUR on the presence of febrile urinary tract infections (*p* = 0.03) with an odds ratio of 6 [CI 95% = (1.13–27.52)].

**Table 3:** Factors associated with risk of fUTI.

	**Univariate model**		**Multivariate model**	
	**OR** **(CI 95%)**	***p*** **-value**	**OR** **(CI 95%)**	***p*** **-value**
Size at birth, cm	1.24 (0.93–1.64)	0.14	/	
Weight at birth, g (per 100g increase)	1.18 (1.02–1.37)	0.03	/	
Age at diagnosis, GA	0.87 (0.75–1.01)	0.07	/	
Presence of VUR	4.33 (0.98–19.20)	0.05	5.57 (1.13–27.52)	0.03
Dysplasia on DMSA scan	2.22 (0.46–10.68)	0.32	/	
Nadir creatinin (≥75 mol/L)	1.01 (0.99–1.04)	0,15	/	

VUR, vesicoureteral reflux.

**Figure 1:**
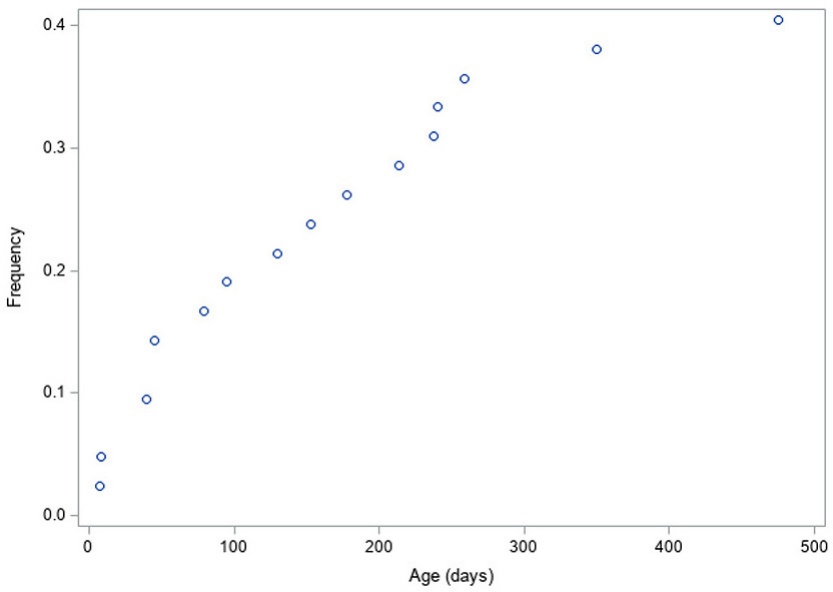
Proportion of patients presenting a fUTI according to age at first fUTI.

As concerns timing of episodes of fUTI, we observed three distinct time periods, with a first decrease in incidence of fUTIs after the first 40 days of life, followed by a second decrease at 240 days of life (Figure 1).

## Discussion

Boys with posterior urethral valves present an increased risk of fUTI. Many children with PUV are put on antibiotic prophylaxis systematically (3). Circumcision status significantly impacts the risk of fUTI as confirmed by the CIRCUP trial (4). The question that comes next is whether all boys with PUV require antibiotic prophylaxis or if some children could do without antibiotic prophylaxis altogether?

There are known risk factors for UTI in the general pediatric population, including gender, circumcision status, younger age, white race, high-grade VUR, CAKUT, BBD, and instrumentation of the urinary tract, but boys with PUV present by definition both BBD and a CAKUT (5). There have been studies on risk factors in children with hydronephrosis, showing again that circumcision status, presence of VUR and lack of continuous antibiotic prophylaxis were risk factors for febrile urinary tract infection (6). The EAU–ESPU guidelines suggest antibiotic prophylaxis might be indicated in boys with high-grade VUR and PUV. However, all the current evidence is not derived from studies specifically on boys with PUV, and even the EAU-ESPU guidelines are supported by a reference to the Swedish reflux study which was not boys with PUV (7, 8).

To this purpose, we took advantage of a large cohort of prospectively followed boys with neonatally diagnosed PUV. This is a homogenous group of boys who underwent valve resection in the neonatal period and were followed, with a standardized protocol, for 2 years. The definition used for qualifying febrile urinary tract infections was strict, and only episodes validated by an independent scientific committee were accepted. They all had control cystograms to exclude the presence of residual leaflets and systematic early DMSA scans. As there was only one event in the circumcision group meaning we would be comparing one patient with fUTI to 48 patients without fUTI, we decided to only study the group without circumcision. This meant comparing 17 children with fUTI to 25 without.

This study shows several interesting points. The first is that presence of dilating VUR is an independent and significant risk factor in this population, with an OR of 6 in our study. This was clearly suspected by many but again, it had not been clearly demonstrated in this population who present other traditional known risk factors (young age, CAKUT, etc.). On the other hand, other variables, such as DMSA scan results or renal function as defined by nadir creatinine, that could have been associated with higher risk of fUTI were not. We did also see three distinct periods for fUTI, during the 1st year of life, with a high-risk period during the first 1–2 months followed by a second period for 6 months and a decrease in risk after 8–9 months of life.

There are limitations to this study. We chose to study only the control group (uncircumcised) of the CIRCUP trial, since the protective effect of circumcision was such that the circumcision group was not exploitable for the purpose of identifying risk factors. We did not take into account specific bladder treatment such as anticholinergics or alphablockers. These patients, under the age of two did not undergo invasive urodynamics. Bladder function was evaluated clinically and using ultrasound parameters such as evolution of upper urinary tract dilatation and post-void residuals and was managed accordingly, but we had insufficient data to characterize bladder function. All our patients were on antibiotic prophylaxis which probably buffers the effect of some risk factors, and we do not have a control group without circumcision nor antibiotic prophylaxis, but we are not sure that this would be ethical knowing the risk of fUTI in this population. Adherence to antibiotic prophylaxis decreased progressively but was equivalent in both groups and does not hinder comparison of the characteristics of children with and without fUTI. Finally, the effect of partially or totally retractable foreskin was not assessed in our study, though defining how retractable the foreskin is can be challenging and tailoring according prophylaxis to variable degrees of retractability is not feasible. The strength of the study is its prospective nature, homogeneous population and strict follow-up protocol.

In conclusion, boys with dilating VUR and PUV are at higher risk of fUTI than boys with PUV alone. The risk of fUTI seems to decrease after 3 and 9 months. Further prospective studies could allow for more precise tailoring of fUTI prevention in this population.

## Data availability statement

The raw data supporting the conclusions of this article will be made available by the authors, without undue reservation.

## Ethics statement

The studies involving human participants were reviewed and approved by Comité de Protection des Personnes Sud-Ouest et Outre Mer III, France. Written informed consent to participate in this study was provided by the participants' legal guardian/next of kin.

## Author contributions

LHa wrote the article. All authors contributed to data collection, analysis, and approved the final version of the article.

## Conflict of interest

The authors declare that the research was conducted in the absence of any commercial or financial relationships that could be construed as a potential conflict of interest.

## Publisher's note

All claims expressed in this article are solely those of the authors and do not necessarily represent those of their affiliated organizations, or those of the publisher, the editors and the reviewers. Any product that may be evaluated in this article, or claim that may be made by its manufacturer, is not guaranteed or endorsed by the publisher.

## Abbreviations

PUV: posterior urethral valves
fUTI: febrile urinary tract infection
DMSA: dimercaptosuccinic acid
RCT: Randomized clinical trial
BBD: bladder and bowel dysfunction
CAKUT: congenital anomaly of the kidney and urinary tract.
